# Characteristics of physician associates/assistants in dermatology

**DOI:** 10.1007/s00403-023-02593-7

**Published:** 2023-03-13

**Authors:** Cynthia F. Griffith, Peter A. Young, Roderick S. Hooker, Kasey Puckett, Andrzej Kozikowski

**Affiliations:** 1 Department of Dermatology, University of Texas Southwestern Medical Center, 5939 Harry Hines Blvd, Dallas, Texas USA; 2 Department of Dermatology, The Permanente Medical Group, Sacramento, California USA; 3 Department of Dermatology, Stanford University School of Medicine, Stanford, California USA; 4 Adjunct Professor Health Policy, Northern Arizona University Biomedical Campus, Phoenix, AZ USA; 5 National Commission On Certification of Physician Assistants, Johns Creek, Georgia USA

**Keywords:** Dermatology Physician Assistant, Physician associate, Dermatologist, Dermatology workforce

## Abstract

The dermatology workforce includes physicians, nurse practitioners (NPs), and physician associates/assistants (PAs). The number of dermatologists is growing slowly while the growth of PAs working in dermatology is rapid and accelerating. To understand their characteristics, a descriptive study of PAs practicing in dermatology utilizing the National Commission on Certification of Physician Assistants (NCCPA) workforce dataset on PA practices was undertaken. NCCPA certifies PAs who practice in the United States and queries them about their role, employment, salary, and job satisfaction. Analyses consisted of descriptive statistics, Chi-Square, and Mann–Whitney tests for comparisons between PAs practicing in dermatology versus the total of all other PA specialties. As of 2021, 4,580 certified PAs reported practicing in dermatology—a nearly twofold increase since 2013, when 2323 worked in the specialty. This cohort's median age was 39 years, and 82% were female. Almost all (91.5%) are office based, and 81% work more than 31 h per week. The median salary was $125,000 (2020 dollars). Dermatology PAs work fewer hours and see more patients than their counterparts compared to all 69 PA specialties. At the same time, dermatology PAs are more satisfied and less burnt out when compared to all PAs. The increased number of PAs selecting dermatology as their discipline can help lessen the projected physician shortage in this field.

## Introduction

Dermatology has been a practice focus of American physician associates/assistants (PAs) since 1980 [1]. To meet growing patient demand for dermatology services due to greater health awareness, market penetration, and an aging population [5, 6], the specialty of dermatology has transitioned to a team-based care delivery model, like many other specialties [2, 3]. This growth and the availability of PAs and nurse practitioners (NPs) may result in dermatology being less affected by workforce shortages than other specialties [4].

The characteristics of PAs working in dermatology are necessary to understand their contribution to the current dermatology workforce. Recent studies show they are predominantly located in urban settings and less racially diverse than PAs overall [7, 8]. Dermatology is the fifth most common PA specialty [9]. We set out to lay the groundwork for future research by characterizing dermatology PAs in more detail.

The research question centers on the following:

*What are the characteristics of dermatology PAs, their practice environments, productivity, and job satisfaction?*

## Methods

Our cross-sectional analyses relied on National Commission on Certification of PAs (NCCPA) health workforce data. This dataset includes demographic and practice characteristics of certified PAs in the US [9]. Initial board certification with NCCPA is required in every state in the United States and the District of Columbia to become licensed to practice medicine. Over 98% of PAs maintain their certification throughout their careers. Therefore this dataset represents most practicing PAs in the US. In addition to administrative data, NCCPA gathers self-reported PA health workforce data via optional algorithm-driven questions in the PA Professional Profile [10]. Items in the PA Professional Profile were developed in 2012 based on the Health Resource and Services Administration's Center for Workforce Studies' minimum data set (MDS) guidelines [11]. American PAs are encouraged to complete or update their information when logging onto the secure website or entering their continuing medical education credits, which is mandatory to maintain certification [12].

NCCPA adheres to various quality control procedures to ensure data quality. Investigators can request de-identified and aggregated PA workforce data for ethical research studies. This study extracted three sets of variables from the PA Professional Profile and then compared the dermatology PA cohort to all PAs in all 69 medical and surgical specialties. The first dataset was demographics, including age, gender, region, urbanity, and language spoken other than English with patients. The second was the following practice characteristics: practice setting, years certified (a proxy for years of PA practice experience), weekly hours worked, patients seen per week in a principal clinical position, whether PAs have a secondary position (non-clinical [education, research, administration] or another clinical position), and if they participate in telemedicine. Further variables explored were income, job satisfaction, burnout, planning to leave the principal clinical position in the next 12 months, and retiring in the next five years.

PAs were asked to provide their gross income from January to December of the prior year from all their PA positions. Response brackets ranged from under $40,000 to over $200,000, in increments of $10,000. We used the midpoint of each income bracket to create a continuous income variable. For statistical analyses, job satisfaction was assessed using a single item with a 7-point response scale ranging from “entirely dissatisfied” to “completely satisfied,” which we dichotomized into satisfied and unsatisfied. A single validated item evaluated burnout with a five-point response scale, then dichotomized into “no symptoms of burnout” and “one or more symptoms,” as in prior studies [13, 14]. PAs were excluded from analyses if they did not review their NCCPA profile in the past three years, were not active clinically, or did not report their practice specialty. This resulted in excluding 29.7% of the 158,470 certified PAs in 2021, for a final analytical sample of 111,428.

Statistical analysis included descriptive statistics (counts and percentages for categorical variables and means [SDs] and medians [IQRs] for continuous measures). As appropriate, bivariate analyses consisting of Chi-Square Tests or Mann–Whitney Tests compared PAs practicing in dermatology versus the total of all other specialties (excluding dermatology) on demographics, practice attributes, and other significant factors of professional characteristics. For all analyses where a comparison was made, a P value of less than 0.05 was considered statistically significant. All analyses were conducted using SPSS.

## Results

At year's end of 2021, 4580 certified PAs reported that they practice in dermatology (almost a twofold increase since 2013, when there were 2,323). This represented 4.1% of the study population.

**Fig. 1:**
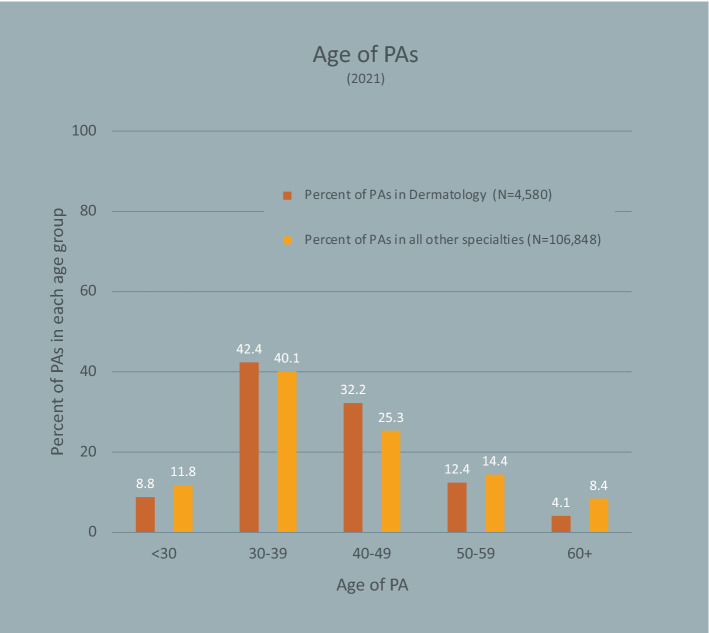
Age Distribution of Dermatology PAs

The mean dermatology PA age was similar to that of other specialties (40.5 vs. 41.4; p = 0.210; Fig. 1). However, a smaller proportion of PAs in dermatology were 50 or older compared to the total of PAs practicing in all other specialties (16.5% vs. 22.8%; p < 0.001).

**Fig. 2:**
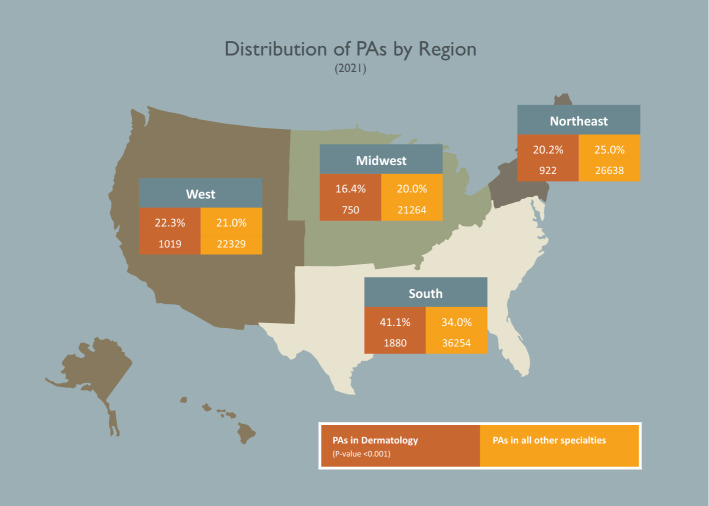
US Regions Where Dermatology PAs Practice

While most PAs are female, this is more pronounced in dermatology than in all other specialties (82.0% vs. 68.6%; p < 0.001). (Table 1) When compared to PAs in all other specialties, PAs in dermatology were more likely to reside in the South US region (41.1% vs. 34.0%; p < 0.001) while being less likely to work in a rural geographic setting (5.8% vs. 7.6%; p < 0.001) and speak a language other than English with patients (18.7% vs. 22.9%; p < 0.001) (Fig. 2).

**Table 1:** Characteristics of Dermatology PAs

	PAs in Dermatology (N = 4580)		PAs in All Other Specialties (N = 106,848)	
	Actual	Percent	Actual	Percent
**Gender (p-value < 0.001)**				
Female	3754	82.0%	73244	68.6%
Male	826	18.0%	33594	31.4%
**Race (p-value < 0.001)**				
White	3806	87.0%	86576	84.7%
Asian	288	6.6%	6129	6.0%
Multi-race	86	2.0%	2131	2.1%
Black/African American	64	1.5%	3627	3.6%
Other^*^	133	3.0%	3697	3.6%
**Ethnicity (p-value < 0.388)**				
Not Hispanic	4110	93.7%	95782	93.4%
Hispanic	277	6.3%	6818	6.6%
**Urban/Rural Setting (p-value < 0.001)**				
Urban	4300	94.2%	98189	92.4%
Large Rural	188	4.1%	4615	4.3%
Small Rural	48	1.1%	2001	1.9%
Isolated	27	0.6%	1508	1.4%
**Speaks a language other than English (p-value < 0.001)**				
No	3600	81.3%	80188	77.1%
Yes	826	18.7%	23760	22.9%

Data as of 2021

*Other includes those who selected “other”, Native Hawaiian/Pacific Islander, and American Indian or Alaska Native.

**Fig. 3:**
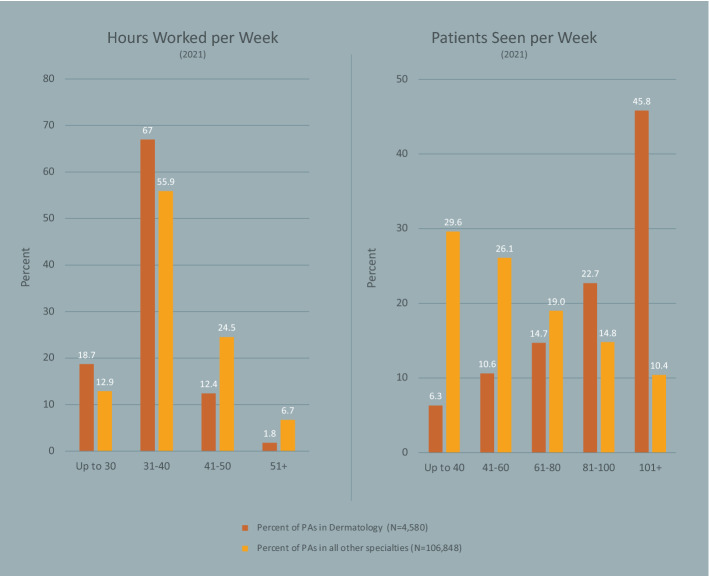
Dermatology PA Productivity

Figure 3 presents results of comparing PAs in dermatology to PAs in all other specialties on practice characteristics. We observed that almost all PAs in dermatology were in office-based private practice settings versus about a third in all other specialties (91.5% vs. 35.1%; p < 0.001). The median years certified (a proxy measure for years of professional PA experience) was slightly longer (11 vs. 10; p < 0.001, respectively). Notably, a much higher proportion of PAs in dermatology versus all other specialties reported seeing 101 or more patients weekly (45.8% vs. 10.4%; p < 0.001); at the same time, PAs in dermatology had a lower proportion working more than 40 h per week (14.2% vs. 31.2%; p < 0.001). PAs in the dermatology discipline were less likely to work in more than one position (8.0% vs. 15.6%; p < 0.001) but had a higher likelihood of providing telemedicine services (39.6% vs. 33.3%; p < 0.001) to their patients when compared to their counterparts.

**Fig. 4:**
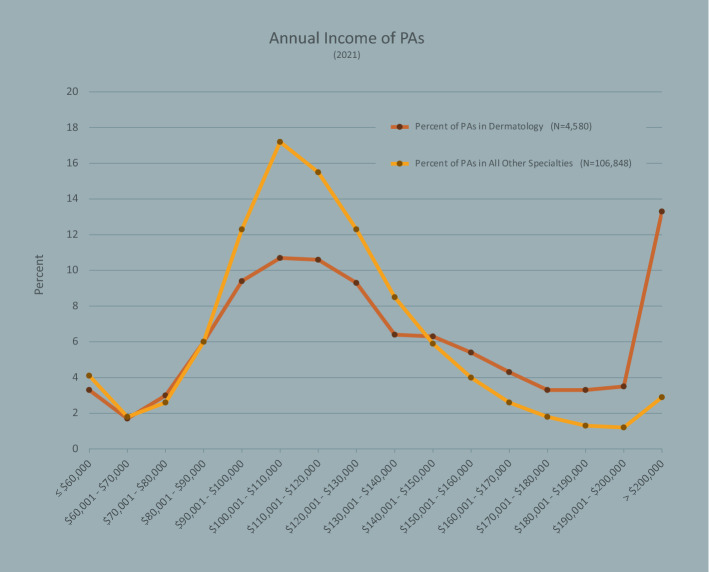
Dermatology PA Annual Income

The total income from PAs practicing dermatology was compared to other PAs (Fig. 4**).** The median income of PAs in dermatology was higher than all other PAs ($125,000 vs. $115,000; p < 0.001). A higher proportion of PAs in dermatology reported earning over $150,000 compared to PAs in all other specialties (33.1% vs. 13.8%; p < 0.001).

**Fig. 5:**
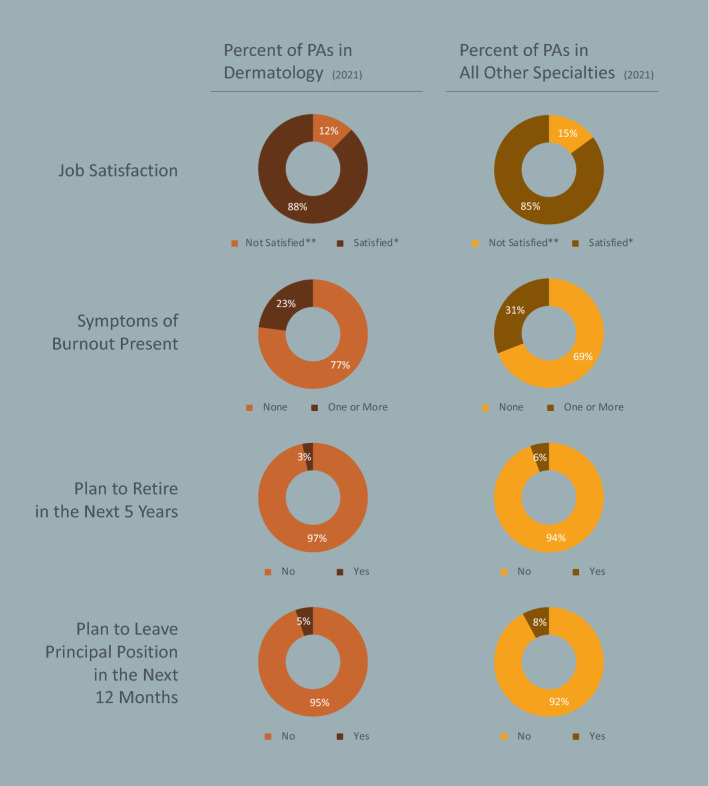
Dermatology PA Job Satisfaction and Career

Job satisfaction of PAs in dermatology is high (87.6% vs. 85.1% for other PAs; p < 0.001), and fewer have any symptoms of burnout compared with PAs practicing in different specialties (23.0% vs. 31.0%, respectively; p < 0.001). Dermatology PAs were less likely to be planning to leave their position in the next year (5.2% vs. 7.9%; p < 0.001) or to retire in the next five years (3.1% vs. 5.5%; p < 0.001) (Fig. 5).

## Discussion

The absolute number of PAs in dermatology is growing during a shortage of dermatologists and a concurrent rise in demand for dermatologic services [5]. As of 2021, 4,580 certified PAs reported practicing in dermatology—almost a two-fold increase since 2013, when there were 2,323. However, in terms of the proportions of the PA workforce practicing in dermatology, it slightly decreased from 4.3% in 2013 to 4.1% in 2021. Most are female (82.0%) and work in offices or settings outside the hospital. This analysis suggests they are in a growth phase and satisfied with their career. The population of PAs in dermatology and their increasing availability enable them to help alleviate the projected physician shortage [15]. It is a relatively young cadre (median age 39 years), female (82.0%), office-based (91.5%), but distributed unevenly throughout the US, with 41.1% in the South. It is unclear if any of the statistical differences such as region, urbanity, language skills, identified in this analysis have clinical implications in a diverse country such as the US.

Knowing whether the PA dermatology workforce is growing along with their demographic and practice characteristics is an essential initial step in understanding how specialized health care is provided to an increasingly older American population. Such clinician data is necessary for planning a society with patients increasingly seeking dermatology services. This new information has numerous ramifications, including more accurate health workforce planning and effective policymaking, and projecting provider surpluses and shortages.

PAs working in dermatology work fewer hours and see more patients than PAs working in all other 69 specialties. They report being more satisfied with their jobs, less burnt out, and less likely to retire in the next five years than their counterparts in other specialties. Areas of future research might elucidate the specific diagnoses, relative value units (RVUs), procedures performed, and prescribing information by PAs in this specialty. Understanding the degree of overlap between dermatology providers (physicians, PAs, NPs) could improve staffing arrangements for employers depending on the needs of the population being served.

High rates of physician retirement or departure from medical practice suggest underlying issues and vacancy challenges for employers [16]. While the information about PAs in dermatology adds to the literature about this group of health professionals, it is unclear whether they are backfilling departed physician roles or being added as an additional staff [8].

The Association of American Medical Colleges reports that there were 12,516 dermatologists in 2020 [20] The Society of Dermatology Nurse Practitioners reports a 2022 membership of 5000 [21]. The numbers and roles of PAs and NPs across the nation have expanded, and reports of their activity are growing at a time when growth of dermatologists is modest [17, 18].

## Limitations and strengths

Our study is not without limitations. The first is the self-reported nature of our data, which is subject to memory or recall bias and misinterpretation of questions. However, recent research suggests that NCCPA health workforce data is reliable and consistent with federal sources [19]. Additionally, direct observation of daily clinical activity and analyzing administrative data using international classification of diseases (ICD) codes would improve the validity of the self-reported specialty practice designation. Several strengths counterbalance these limitations. This study draws upon the most comprehensive national dataset on certified PAs: the PA Professional Profile, which was developed based on Minimum Data Set guidelines. Future research could contextualize our results with other data sources, including the US Census, Bureau of Labor Statistics, and American Community Survey.

## Conclusion

Understanding PA and NP characteristics and employment settings are essential for research on health workforce supply and demand. Our analysis revealed that the supply of PAs in dermatology is increasing. These relatively young, female, office-based PAs work fewer hours and see more patients than their counterparts. At the same time, they are more satisfied and less burnt out. With this groundwork on the modern PA dermatology workforce, further research should investigate the economics of their labor, clinical team attributes, patient outcomes, and satisfaction.

## Data Availability

The data underlying this article cannot be shared publicly due to the privacy of individuals participating in the study. The data will be shared on reasonable request with the corresponding author.
